# A novel mechanical chest compressor with rapid deployment in all population cardiopulmonary resuscitation

**DOI:** 10.1038/s41598-020-63058-9

**Published:** 2020-04-08

**Authors:** Chih-Wei Sung, Hung-Chih Wang, Jiann-Shing Shieh, Fu-Shan Jaw

**Affiliations:** 1 Institute of Biomedical Engineering, National Taiwan University, Taipei, Taiwan; 2 Department of Emergency Medicine, National Taiwan University Hospital Hsin-Chu Branch, Hsinchu, Taiwan; 3 Department of Mechanical Engineering, Yuan Ze University, Taoyuan, Taiwan

**Keywords:** Biomedical engineering, Mechanical engineering

## Abstract

Cardiopulmonary resuscitation (CPR) resuscitates patients suffering from cardiac arrest. Mechanical chest compression CPR highlights the need for high CPR quality to facilitate survival and neurological recovery. However, current CPR devices cannot be used on pregnant women or infants. These devices’ long re-setup times interrupt CPR and can cause cerebral ischemia. This study designed a novel device with a crank-sliding mechanism. The polar coordinate system (r, θ, z) shortened the setup time and enabled adjustment without moving the patient. We compared our device with commercial products (e.g., LUCAS-2) by quantifying the compression pressure. Control groups for manual CPR of trained physicians and untrained citizens were recruited. We used Resusci Anne products as models. Our results indicated that our design exhibited performance similar to that of LUCAS-2 in adults (557.8 vs. 623.6 mmHg, *p* = 0.217) and met the current CPR standard guidelines. Notably, our device is applicable to pregnant women [565 vs. 564.5 (adults) mmHg, *p* = 0.987] and infants [570.8 vs. 564.5 (adults) mmHg, *p* = 0.801] without lowering the compression quality. The overall compression quality and stability of mechanical chest compression CPR were favorable to those of manual CPR. Our device provides an innovative prototype for the next generation of CPR facilities.

## Introduction

Out-of-hospital cardiac arrest is a global public health concern, with 420,000 cases in the United States, 275,000 cases in Europe, and 220,000 cases in Asia documented annually^1–3^. Rates of survival to hospital discharge range between 5% and 20%^2^. Survivors may experience severe neurological sequela even following receipt of sustained return of spontaneous circulation (ROSC) after cardiopulmonary resuscitation (CPR), and this increases medical costs and decreases patients’ quality of life after hospital discharge. The quality of CPR is key to resuscitation success and long-term prognosis. High-quality CPR, as defined by the American Heart Association and European Resuscitation Council, involves four aspects: a high compression rate (100 to 120 per minute), sufficient compression depth (5 cm in adults and teenagers; 4 cm in infants), full chest recoil, and minimal hands-off time^4,5^. Table 1 lists CPR requirements in multiple populations.

**Table 1:** 2015 CPR Guidelines of the European Resuscitation Council and American Heart Association [4,5].

Component	Adults and Teenagers	Children	Infants
Compression rate	100–120/min	100–120/min	100–120/min
Compression depth	5 cm	5 cm	4 cm
Hand placement	Two hands on the lower half of the sternum	Two hands on the lower half of the sternum	Two fingers in the center of the chest
Chest recoil	Allow full recoil of the chest after each compression; do not lean on the chest after each compression		
Interruption minimization	Limit interruptions in chest compressions to less than 10 seconds		

To deliver high-quality CPR, manual chest compression CPR (M-CPR) is traditionally the initial tool used for resuscitation. However, various problems with M-CPR can reduce CPR quality, including insufficient compression depth, compression rate variation and consequential low mean arterial pressure, diastolic arterial pressure, and coronary perfusion pressure^6^. Even the highest-quality M-CPR provides only 20%–30% of the usual cardiac output and thus may considerably reduce the ROSC rate or lead to negative neurological outcomes^7^.

Recently, the use of mechanical chest compression CPR (Mcc-CPR) has shed light on various aspects related to CPR, including emergency medical services at scenes of accidents, transportation, and resuscitation room scenarios. Mcc-CPR–assisted facilities have certain advantages over M-CPR; Mcc-CPR devices can provide a stable compression depth and frequency over a long period in a variety of clinical scenarios such as those involving up-downstairs, ambulances, and patient posture^8,9^. Several studies involving adult animal models and humans have shown that Mcc-CPR has the advantage of relatively long CPR duration in critical resuscitation environments^10,11^. One meta-analysis further suggested that Mcc-CPR could be used to deliver chest compressions when doing so through M-CPR is difficult or impossible^12^.

Current commercial machines use an automatic piston (Thumper^®^, Michigan Instruments, Kentwood, MI, USA; LUCAS-2^®^, Physio-Control/Jolife AB, Lund, Sweden) or band-like mechanism (AutoPulse^®^, Zoll Medical Corporation, Chelmsford, MA, USA)^13^. LUCAS-2 uses a piston to compress a patient’s chest wall through continuous simple harmonic motion (SHM) and is supplied by a motorized electric power source. Potential reaction force is compensated for by symmetric arms. The piston by Thumper is driven by compressed air, and the compression rate and depth can be adjusted by scales. By contrast, AutoPulse, a load-distributing band device, operates through a wide band that fits around the chest, whose circumference is alternately shortened and lengthened to provide rhythmic chest compressions. Although these three devices provide safe, efficient CPR assistance, some limitations restrict their deployment in some resuscitation situations.

First, the aforementioned commercial devices have been approved for use on adults only and cannot operate at tilted angles; they have not been tested for use on pregnant women. This is problematic because a 30° tilt toward the left side of the body during CPR facilitates blood return and can prevent inferior vena cava syndrome in pregnant women suffering from cardiac arrest. In addition, in the aforementioned commercial devices, the compression depth and piston size cannot be altered, and thus these devices cannot be used on infants. Second, if a patient lies in such a position that his or her heart cannot receive optimized compression, the re-setup time required necessitates the discontinuation of CPR, which prolongs the cerebral ischemic time and increases the likelihood of negative outcomes. When LUCAS-2 or Thumper is used, the rescuer can move the patient but cannot move the device because of limitations in the mechanical architecture. When AutoPulse is used, the band must be loosened and then tightened again after the patient has been moved. Moving an unconscious patient requires time and increases the demand for manpower.

In this study, we designed a novel mechanical chest compressor with an exchangeable piston and alterable compression depth that is suitable for use on adults, infants, and pregnant women. We also designed a new mechanical structure that converts Cartesian coordinates into polar coordinates to reduce the setup time, thereby shortening the aforementioned discontinuation period.

## Materials and Methods

### Mechelper structure

**Figure 1:**
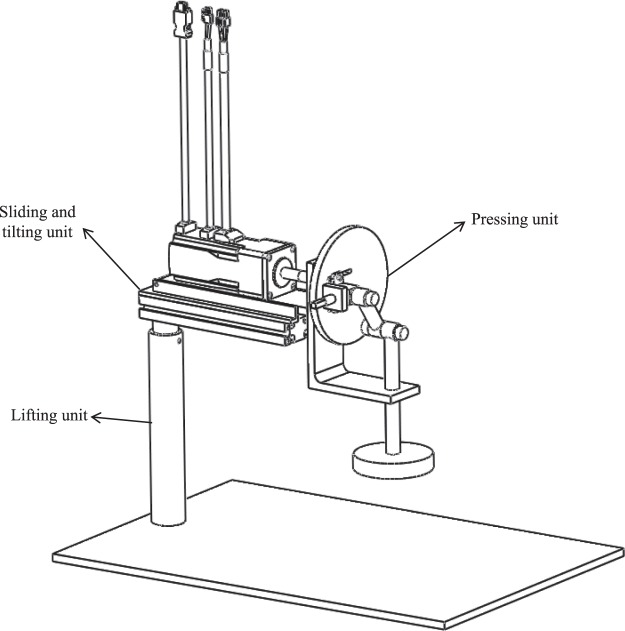
Schematic of the whole structure.

The proposed design, named “Mechelper,” has three main parts: a pressing unit, sliding and tilting unit, and lifting unit. Figure 1 presents a schematic developed using SolidWorks^®^ 3D CAD (Dassault Systèmes, SolidWorks Corp., Waltham, MA, USA).

### Pressing unit

**Figure 2:**
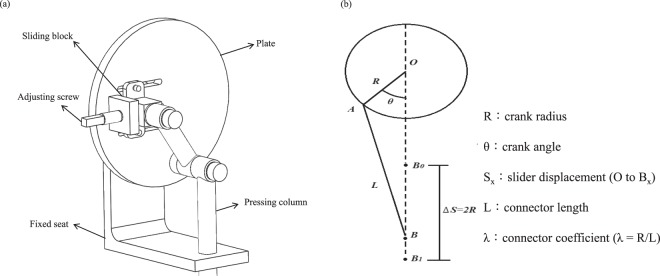
Schematic of (**a**) pressing unit; (**b**) mechanical model of the crank-sliding structure.

The pressing unit (Fig. 2(a)) transforms the rotation motion into a linear reciprocating motion that acts as SHM. The core component is the crank-sliding mechanism, which comprises a crank, connector, sliding block, and sliding rail. This mechanism not only provides SHM but also adjusts the compression depth by extending the crank length; the principle is shown in Fig. 2(b).

The distance *S* between the center of the plate and the slider is expressed as follows:

1 $$S=R\,[(1-\,\cos (\theta )+\frac{\lambda }{2}{\text{sin}}^{2}(\theta )]$$

2 $$\begin{array}{ccc}{S}_{B1}-{S}_{B0}=R\,\left[1,-,\,,\cos ,(,{180}^{\circ },),+,\frac{\lambda }{2},{\sin }^{2},(,{180}^{\circ },)\right]-R\,[(1-\,\cos ({0}^{\circ })+\frac{\lambda }{2}{\sin }^{2}({0}^{\circ })] &  & \\ =\,R\,\left[1,-,(,-,1,),+,\frac{\lambda }{2},\times ,0\right]-R\,[(1-1)+\frac{\lambda }{2}\times 0] &  & \\ =\,2R &  & \end{array}$$

Therefore, the compression depth (distance between B_1_ and B_0_) is twice the crank radius *R*. By adjusting the length *S*, the adjusting screw forces the Mechelper to switch on two modes (for adults and infants). When the device is activated, the plate rotates. The compression depth is determined by the length between the sliding block and the center of the plate. The pressing column is restricted by the fixed seat and performs SHM. Therefore, Mechelper can be used on both adults and infants.

The terminal component of the pressing column is the changeable compression pad. In adult resuscitation, the rescuer crosses his or her hands, places them on the center of the nipple line, and then compresses the chest wall. The average covering diameter of an adult’s palm is 7.9 cm. Thus, we selected a compression pad with a diameter of 8 cm for adults. In infant resuscitation, the rescuer overlaps the distal phalanx and applies compression at the center of the nipple line. The average compression diameter is 3 cm; we used this diameter for the compression pad for use on infants. The quick-coupling parts are located between the compression pads and pressing column. The quick-coupling parts that we used were Hi-Cupla 20PM and Hi-Cupla 20SM (Nitto Kohki Co., Ltd.), both of which feature a two-sided screw, robust structure, and rapid switch (less than 2 seconds).

### Sliding and tilting unit

**Figure 3:**
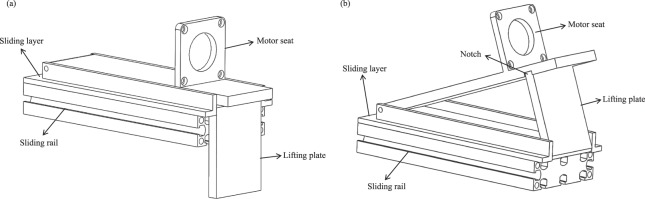
Schematic of (**a**) the sliding and tilting unit; (**b**) lifting unit.

Figure 3(a,b) depict the sliding and tilting unit before and after tilting, respectively. We designed robotic arms with multiple functions that are flexible in their ability to replace the Cartesian coordinate system (x, y, z) with the polar coordinate system (r, θ) in order to reduce the setup time. First, when the patient is a pregnant woman, the lifting plate is embedded into a notch, yielding an angle of 30°. Second, the slider mechanism contains a slider layer and a slider rail. To extend the operating range in the polar coordinate system (r), a handle screw is used to fasten the slider layer to the slider rail. When the screw is loosened, the rescuer can adjust the position of the slider layer. When the screw is tightened, the slider layer has a fixed position on the slider rail.

### Lifting unit

Body shape and the anterior-to-posterior diameter of the thoracic cavity vary among patients. In the polar coordinate system (r, θ, z), adjustment of the z-axis is essential. We developed a two-column design with an inner column and outer column. The outer column has a hole at the top. The inner column is placed inside the outer column, which can move up and down. A handle screw is placed in the hole to fix the inner column. Once loosened, the inner column can move easily. In this design, the z values ranged from 8 to 30 cm.

Additionally, because of its hollow aluminum columns, this mechanism enables rescuers to freely shift the position of the pressing column in order to adjust θ in the polar coordinate system. In other words, the values of z and θ can be modified simultaneously.

### Power sources

In this study, a stepper motor, namely a brushless direct current electric motor, was used to provide input power. Compared with a servomotor or gas cylinder, this stepper motor has the advantages of precise position control, easy operation, relatively low volume, and light weight. The AZM66MK-PS1 (Taiwan Oriental Motor Co., Ltd., New Taipei City, Taiwan) stepper motor was used in this study.

### Sensors, testing environment, and protocol

Resusci Anne ZY-CPR 100 and Resusci Anne ZY-CPR 140 were used to simulate an adult and infant, respectively. We performed serial CPR on an adult, a pregnant woman, and an infant. Each CPR session lasted 2 minutes. Notably, we recruited M-CPR groups consisting of physicians (n = 3, more than 2 years’ emergency department training) and citizens (n = 3, untrained, age matched: 28 to 32 years old) as reference groups. Mechelper, LUCAS-2, the physicians, and the citizens each performed 2 minutes of CPR twice on Resusci Anne ZY-CPR 100 (adult) sequentially. Afterwards, another two scenarios (pregnant woman and infants) operated in the same steps. In other words, for each subject, he has to perform the M-CPR in different conditions [with normal angle in adults, with a tilt setting (pregnant woman), and with Resusci Anne ZY-CPR 140 (infant)], twice in each condition. However, the pregnant woman and infant not administered CPR by LUCAS-2 due to its limitation.

The CONFORMat^TM^ system (Tekscan Inc., Boston, MA, USA) was used to quantify the quality of CPR chest compression. This system provides accurate, real-time information regarding pressure distribution and the center of the force trajectory. During each 2-minute CPR session, the pressure of each compression was detected. Subsequently, sensors sent this raw data to a microprocessor, amplified the signal, and then digitized and extracted the data through a USB interface. Output pressure was expressed in millimeters of mercury (mmHg).

In setup time simulation, another 3 independent nurses (1 to 2 years in the hospital) were recruited in the simulation. The physicians, nurses and citizens deployed the both Mechelper and LUCAS-2 system twice, respectively. The deployment time was defined as the duration between receiving order and the first successful chest compression. The adjustment time was defined as the time form the artificial placement in error region to the first successful chest compression. The average with standard deviation was calculated.

Additionally, to demonstrate the chest wall recoil property, Mechelper and other rescuers performed upon the real-time audio-visual CPR feedback provided by a defibrillator/monitor (ZOLL R Series, ZOLL Medical Corporation, Chelmsford, MA). A compression elastic recoiling indicator provides the real-time chest wall recoil. When the bar shows full, it implies that the operators have come completely off the chest to allow for full elastic recoil.

### Statistical analysis

Continuous variables with normal distributions were presented as means ± standard deviation and were compared among the groups by conducting a one-way analysis of variance (ANOVA). The Bonferroni method was used as a post hoc test after ANOVA. Statistical significance was set at *p* ≤ 0.05. Statistical analyses were conducted using SAS (version 9.4, SAS Institute, Chicago, IL, USA).

## Results

### Operation on adults

**Figure 4:**
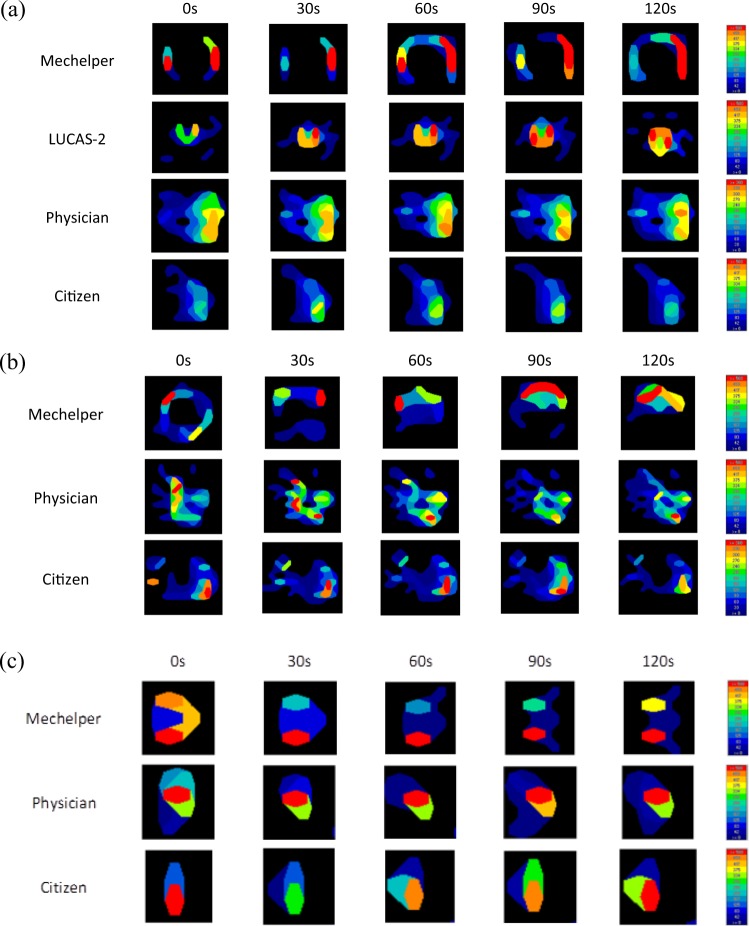
Illustration of compression pressure (mmHg) over time between our device and other rescuers throughout 2 minutes of chest compression: (**a**) adult mode; (**b**) pregnant woman mode; (**c**) infant mode.

**Figure 5:**
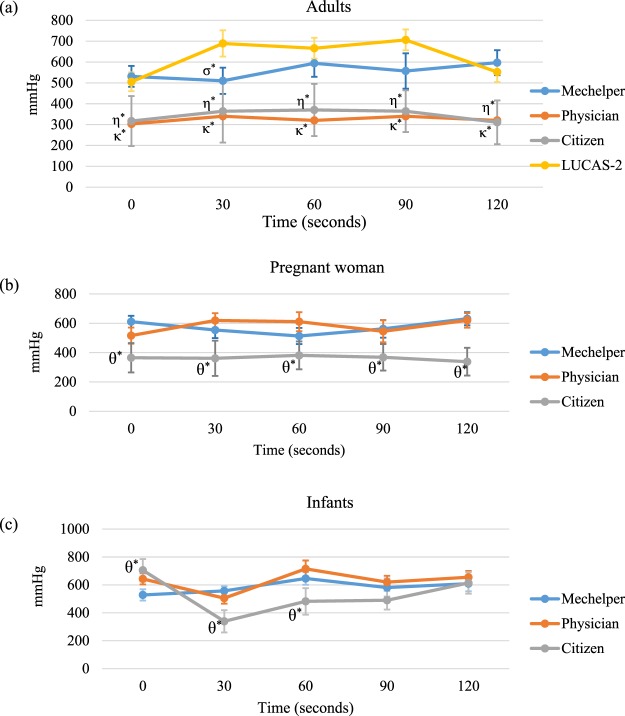
Comparison of compression pressure (mmHg) over time between our device and other rescuers throughout 2 minutes of chest compression: (**a**) adult mode; (**b**) pregnant woman mode; (**c**) infant mode. Bonferroni post hoc test was used to compare each group in each time point. σ*: significant difference as compared with LUCAS-2 (p < 0.05). η*: significant difference as compared with LUCAS-2 (p < 0.05). κ*: significant difference as compared with LUCAS-2 (p < 0.05). θ*: significant difference as compared with Mechelper (p < 0.05).

Figure 4(a–c) demonstrate the operation of Mechelper on adults, pregnant woman and infant. All figures present is a representative image in one trial. For adults, a testing protocol was applied and compression pressure was measured, as shown in Fig. 4(a). In a 2-minute testing period, the average pressure of Mechelper was 557.8 ± 38.2 mmHg, that of LUCAS-2 was 623.6 ± 89.5 mmHg, and those of the physicians and citizens were 324.6 ± 15.7 mmHg and 345.2 ± 28.7 mmHg, respectively. Under consideration of initial instability, we also took measurements during the first compression; the average pressure was 564.5 ± 40.6 mmHg for Mechelper, 653.3 ± 69.5 mmHg for LUCAS-2, 330.0 ± 11.5 mmHg for the physicians, and 352.3 ± 27.6 mmHg for the citizens. Mcc-CPR demonstrated sufficient compression pressure and greater stability than M-CPR (*p* < 0.05). The operating performance of Mechelper was similar to that of LUCAS-2. Average pressure versus time results are shown in Fig. 5(a).

### Operation on pregnant women

Because LUCAS-2 is contraindicated for pregnant women, we measured the compression pressure of Mechelper and M-CPR, as illustrated in Figs. 4(b) and 5(b). The average pressure for Mechelper was 574.2 ± 47.1 mmHg and thus not significantly different from the corresponding result for operation on adults (*p* < 0.05). The physicians exhibited an average pressure of 582.2 ± 48.4 mmHg, and that exhibited by the citizens was significantly lower (362.6 ± 15.7 mmHg, *p* < 0.05). After the first compression results had been disregarded, the average pressure was 565.0 ± 49.0 mmHg for Mechelper, 598.8 ± 36.1 mmHg for the physicians, and 362.0 ± 18.0 mmHg for the citizens. In the tilt resuscitation scenario, Mechelper demonstrated excellent performance; this observation was consistent with that of the normal resuscitation condition.

### Operation on infants

Figure 4(c) depicts the simulations of CPR for infant resuscitation by Mechelper, the physicians, and the citizens. Analysis revealed that the average compression pressure of Mechelper was 562.2 ± 33.3 mmHg, that of the physicians was 627.6 ± 77.0 mmHg, and that of the citizens was 526.0 ± 139.6 mmHg. In the infant resuscitation scenario, M-CPR exhibited unstable performance compared with Mechelper (*p* < 0.05), as shown in Fig. 5(c). LUCAS-2 was also not used for infant resuscitation.

### Operation comparison for long time

**Figure 6:**
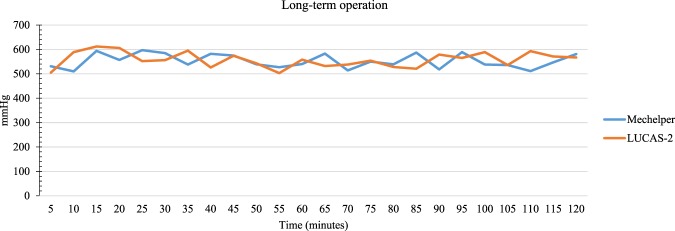
Comparison of compression pressure (mmHg) between Mechelper system and other rescuers throughout 120 minutes of chest compression.

Since a durable system is essential in real CPR scenario, Fig. 6 showed the comparison of the current design and LUCAS-2 to show the efficacy and safety of Mechelper system. The system demonstrated constant, stable and consistent output in chest compression which the compression pressure was between 500 mmHg to 600 mmHg in the 2-hour duration. The physicians and citizens were not included because of undoubtedly failure in long-term chest compression.

**Figure 7:**
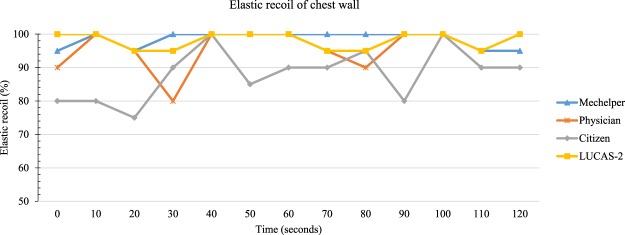
Comparison of chest recoil property among different rescuers.

Figure 7 demonstrated the property of elastic recoil in the CPR among Mechelper, LUCAS-2, physicians and citizens during a 2-minute period. Mcc-CPR exhibited nearly perfect property as compared with M-CPR. Also, Mechelper and LUCAS-2 did not present any significant difference in the duty cycle.

### Setup time simulation

Table 2 demonstrated the setup time comparison between Mechelper and LUCAS-2 system stratified by operators. The average initial setup time (deployment time) of Mechelper was 48.3 seconds, whereas that of LUCAS-2 was 39.1 seconds; this difference was not significant (*p* = 0.103). However, in the re-setup test, the average time (adjustment time) of Mechelper was significantly shorter at 15.4 seconds; this was shorter than that of LUCAS-2 (36.1 seconds, *p* < 0.001). The primary reason for this difference was the need to unlock LUCAS-2, move the patient, and then redeploy the instrument. By contrast, in Mechelper, the compression can be simply adjusted on the basis of the polar coordinate system without moving the patient.

**Table 2:** Comparison of setup time between systems.

	Mechelper	LUCAS-2	*p*
**Deployment time (seconds)**			
Physicians	42.7 (8.7)	36.1 (9.9)	
Nurses	44.1 (9.9)	38.2 (8.6)	
Citizens	58.1 (14.3)	43.0 (11.9)	
Average	48.3 (10.2)	39.1 (11.6)	0.103
**Adjustment time (seconds)**			
Physicians	13.2 (4.4)	27.3 (6.7)	
Nurses	12.9 (5.6)	31.3 (8.9)	
Citizens	20.1 (8.1)	49.7 (12.3)	
Average	15.4 (6.5)	36.1 (8.6)	<0.001

Data are presented as mean (standard deviation).

### Detailed mechelper specifications and comparison

Table 3 lists the detailed specifications of Mechelper and commercial Mcc-CPR products for comparison. Mechelper is 60 cm in length, 52 cm in width, and 70 cm in height and covered with an aluminum alloy. Its total weight is 9.8 kg, and its power is 62 W, supplied by an alternating current of 110 V. The maximum anterior–posterior (AP) diameter of the thoracic cavity is 30 cm. Mechelper is restricted by no limitations regarding patient body mass index. In contrast to some existing commercial products, Mechelper can be used on pregnant women and infants for general resuscitation. Additionally, its setup process is convenient for rescuers.

**Table 3:** Specifications of Mechelper and other products for comparison.

Item	Mechelper	LUCAS-2	Thumper	AutoPulse
Mechanism	piston	piston	piston	loading band
Size (cm)	60 × 52 × 48	57 × 52 × 54	48 × 56 × 23	83 × 45 × 8
Weight (kg)	9.8	7.8	8.6	9.3
Power source	electric	electric	compressed gas	electric
APD (cm)	8–30	17–30	N/A	N/A
Thoracic weight (cm)	no limitation	<45	no limitation	no limitation
Compression depth (cm)	4/5 (two modes)	5.3 ± 0.2	0–8	20% of AP
Compression frequency	100–120	100	100 ± 6	80 ± 5
Setup time (seconds)	25–50	20–40	N/A	N/A
Patients	no limitation	only adults	only adults	only adults

APD = anterior–posterior diameter.

## Discussion

This study designed and implemented a novel mechanical chest compressor with rapid deployment. The proposed compressor not only achieved relatively short setup and re-setup times by converting the traditional Cartesian coordinate system into the polar coordinate system but also can be used on any cardiac arrest patient, including infants and pregnant women, because of its innovative crank-sliding mechanism. For several decades, there has been consensus that high-quality CPR must be available for patients undergoing out-of-hospital cardiac arrest. The worldwide governments aggressively promote public education concerning basic life support and education for medical professionals regarding advanced cardiac life support. However, the current 2015 CPR specifications (Table 1) are seldom met; the compression depth of M-CPR is frequently too shallow and varied, compression rates are often excessive, and prolonged interruption occurs regularly. Mcc-CPR compensates for the drawbacks of M-CPR in prolonged CPR. LUCAS-2^®^, Thumper^®^, and AutoPulse^®^ are devices approved by the Food and Drug Administration of the United States that exhibit excellent performance in CPR; however, some limitations of these devices can reduce their accessibility in clinical situations. Our Mechelper design overcomes these limitations and demonstrates similar efficacy compared with the aforementioned commercial products (Table 3).

Our results revealed that the proposed Mechelper device exhibited a pressure pattern similar to that of LUCAS-2. The distribution of pressure remained constant over time (Fig. 4(a)), although the shape changed. The main reason for this change was the material of the compression pad, which was covered with a bare aluminum alloy; by contrast, the compression pad in LUCAS-2 is covered by a rubber suction cup, which enables a certain degree of compliance at maximum compression so that the maximal force is exerted toward the center. The aluminum alloy was rigid that maximal force would be exerted around the circumference of the pad. Notably, the compression depths measured by the spring of Resusci Anne ZY-CPR 100 for Mechelper and LUCAS-2 were identical (5 cm in adults) (data not shown). Additionally, the line chart in Fig. 5(a) illustrates stability throughout a 2-minute session of chest compression. Whether conducted by trained physicians or untrained citizens, M-CPR failed to exert sufficient pressure and therefore had insufficient compression depth in the dummy model. Generally, as the duration of CPR is prolonged, M-CPR exhibits increasingly unstable CPR performance because of muscle fatigue, even when the compression pressure is constant for 2 minutes.

Pregnant women and infants are clinically vulnerable populations. Although rare, maternal cardiac arrest remains a concern and can be devastating, often resulting in the death of the mother and/or neonate^14^. In a small retrospective study, liver lacerations requiring intervention occurred in 43% of gravidas patients who had survived CPR; this rate was considerably higher than other published rates (0.6–2.1%) among the general patient population^15^. Current commercial devices are contraindicated for pregnant women. However, Mechelper overcame existing limitations through its 30° tilting structure. Our results revealed no significant differences between the average compression pressure for adults and that for pregnant women (564.5 vs. 565 mmHg, *p* = 0.987). Furthermore, Mechelper exhibited excellent compression performance in both perpendicular and tilting environments, suggesting that this device may be applicable for use on patients who cannot lie horizontally because of trauma (e.g., a knife inserted into the posterior back). The aforementioned tilting structure enabled Mcc-CPR for usual cases. In addition, Mechelper exhibited higher stability than M-CPR, for which CPR performance varied significantly between the physicians and citizens (582.2 vs. 362.6 mmHg, *p* < 0.001). Similarly, our device can be used for infant resuscitation. No significant differences were observed between the average compression pressure in infants and that in adults (570.8 vs. 564.5 mmHg, *p* = 0.801) or between that in infants and that in pregnant women (570.8 vs. 565 mmHg, *p* = 0.812). By contrast, M-CPR administered by physicians to infants exhibited relatively great variation in compression pressure during 2 minutes of resuscitation. Such variation may be due to infant tissue. The AP diameter is relatively short in infants, and the resistance of the skin, soft tissue, and even organs is quite different to that of adults. Another influential factor was overlapping thumbs, the use of which fails to provide stable performance compared with the cross-palm method. Therefore, Mcc-CPR exhibited particular significance for infant CPR.

Interestingly, the low pressure was obtained for trained physicians in performing CPR in adults (324.6 mmHg) which was significantly lower than that in pregnant women simulation (582.2 mmHg). It might originate from the self-compensation mechanically. The trained physicians in the hospital performed more than 50 M-CPRs annually. In a general adult scenario, the compression force generates from the lower limb or lower trunk, deliver to central trunk and bilateral shoulders and then deliver to patients via bilateral upper limbs (arms, forearms, and hands). Actually, the arm does not exert one’s efforts but deliver the compression force from the trunk to patient’s thoracic cavity. However, in the pregnant women mode, the force delivery differs. Due to failure or inefficiency of force delivery from trunk to arm, physicians may tend to use their biceps or triceps to generate higher compression pressure for achieving the same CPR quality (depth). Furthermore, the compression force was different between physicians and the mechanical device in adults’ mode (Fig. 5(a)). The mechanism of Mcc-CPR is to generate a fixed compression depth by AC power that contribute a higher compression pressure without doubt. The physicians could perform the optimized force delivery by previous experience. The lower compression with the same compression depth was therefore shown. Of course, M-CPR fails to maintain long-term high quality CPR.

CPR duration is an independent factor in outcome prediction. Longer CPR duration leads to less favorable survival and neurological outcomes^16^; a longer duration of CPR corresponds to a higher in-hospital mortality rate. The primary reason for this is recurrent adjustment to optimize a patient’s position. Another study revealed that Mcc-CPR had marked survival benefits for patients compared with M-CPR when the chest compression duration was longer than 16.5 minutes^17^; this finding reflected the poor quality and potential for fatigue during prolonged periods of human resuscitation. Setup time is crucial; shorter deployment time reduces the overall duration of CPR. A LUCAS-2 report suggested that if the application time was less than 10 seconds, the likelihood of success was greater than 98% in the study population^18^. Device weight, structural complexity, and operator influenced the setup time. Most notably, adjustment during resuscitation led to discontinuation of CPR, increased duration of CPR, or worsened prognoses because of the longer cerebral ischemic time. In the emergency room, after a patient had been moved to the resuscitation bed, Mcc-CPR was deployed immediately, and then CPR was initiated. If the compression position (e.g., the stomach or upper lung) was incorrect, adjustments needed to be made. Moving some patients requires excessive time if they have a heavy weight (especially close to 100 kg). Our device can modify the compression position without moving the patient. By contrast, the two arms and inverse-U shape structure of LUCAS-2 restrict its degree of freedom, and thus it cannot make adjustments in multiple directions simultaneously.

We expect that customized CPR will be a trend in the future because it can increase the efficacy of CPR while minimizing risks during chest compression. This is because the anatomical position, sternum or chest dimensions, and etiology of cardiac arrest differ among patients. In a study involving a swine model, an end-tidal CO_2_-guided (ET-CO_2_) automated robot CPR system was developed and verified as effective^19^. An algorithm was able to find the optimal compression position through real-time ET-CO_2_ feedback; thus, this robot CPR system may be a promising alternative to M-CPR. In clinical trials, sonography-guided assisted Mcc-CPR may contribute to higher CPR quality and provide real-time cardiac output feedback during chest compression.

Customized CPR can also minimize complications that can lead to further negative outcomes after ROSC. Clinically, resuscitation-related rib and sternum damage are the most frequent complications; almost 60% of patients sustain rib fractures during M-CPR. One study observed rib fracture rates of 45% and 39% for LUCAS-2 and AutoPulse, respectively^20^. In addition, some life-threatening conditions such as tension pneumothorax, pneumomediastinum, myocardial injury, and liver rupture during CPR may indirectly lead to in-hospital mortality iatrogenically^21^, and inappropriate compression force or pressure can cause rib fracture or soft tissue injury. The sensor on the compression pad may detect dangerous signals and remind rescuers to discontinue the mechanical compressors between the myocardium and skeletal structure, which are quite different. Furthermore, inappropriate chest compression positions can cause fractures, and thus negative feedback regarding such positions is required. The skin conductance sensor may detect the skin parameter upon and during compression and then analyzed what the tissue is, which distinguishes that whether the heart or the rib is compressed. The optimal CPR quality would be achieved by the customized CPR in the coming era.

## Conclusion

The novel device developed in this study enables rescuers to perform Mcc-CPR on all types of patients. Adjustment based on the polar coordinate system can correct and optimize the compression position without requiring the patient to be moved, thereby shortening the recurrent setup time and minimizing the discontinuation of CPR. In the future, more robust negative feedback systems operated by sensors should be investigated and implemented to further increase the quality and safety of CPR.
